# Successful mesh plug repair using a hybrid method for recurrent inguinal hernia after laparoscopic transabdominal preperitoneal approach: A case report

**DOI:** 10.1016/j.ijscr.2019.05.017

**Published:** 2019-05-10

**Authors:** Rina Kikugawa, Shingo Tsujinaka, Sawako Tamaki, Tsutomu Takenami, Ryo Maemoto, Rintaro Fukuda, Nobuyuki Toyama, Toshiki Rikiyama

**Affiliations:** Department of Surgery, Saitama Medical Center, Jichi Medical University, 1-847, Amanumacho, Omiya, Saitama-shi, Saitama, 330-8503, Japan

**Keywords:** Recurrent inguinal hernia, Mesh plug, Hybrid method

## Abstract

**Introduction:**

The HerniaSurge Group and the European Hernia Society guidelines recommend an anterior approach to treat recurrent inguinal hernias after a failed posterior approach. The hybrid method combining explorative laparoscopy and anterior open approach can provide the benefits of both approaches.

**Presentation of case:**

A 79-year-old man presented with a recurrent inguinal hernia after primary repair for an indirect hernia using the laparoscopic transabdominal preperitoneal approach (TAPP) 5 years ago. The indirect hernia formed inferior to the lower edge of the previous mesh was diagnosed under laparoscopy. The hernia defect (2 cm) was fixed using a mesh plug via the anterior approach. Appropriate mesh overlap was confirmed using laparoscopy.

**Discussion:**

This minimally invasive method enabled us to choose the best treatment for recurrent hernia and prevent chronic pain due to possible nerve damage caused by extended dissection of the scar tissue. Furthermore, the final confirmation step using laparoscopy assures complete coverage of all defects within the myopectineal orifice.

**Conclusion:**

This hybrid method facilitates the choice of an optimal approach for the treatment of recurrent hernia and may reduce surgical complications and re-recurrence rate.

## Highlights

• An anterior approach to inguinal hernia repair using laparoscopy is demonstrated.
• This approach provides added advantage to recurrent inguinal hernia repair.
• This approach can prevent nerve damage by reducing surgical dissection.
• Laparoscopic confirmation assures complete coverage of all hernia defects.

## 1 Introduction

Laparoscopic repair of inguinal hernias has many potential benefits compared with conventional repair [1]. It is logical to infer that there might be an even greater benefit for patients undergoing laparoscopic repair of recurrent inguinal hernias [2,3]. First, the laparoscopic approach enables surgeons to identify the hernial orifice defect, which may decrease the relatively high re-recurrence rate compared to that on primary repair. Moreover, choosing a tailored approach using laparoscopy would be a reasonable option because the potential risk of complications in open recurrent inguinal hernia repair is much higher than that in primary repair.

According to Bisgaard [4], the HerniaSurge Group moderately recommends laparoendoscopic inguinal hernia repair for recurrent hernias after failed anterior or Lichtenstein repairs, where the entrance point is not the same as the previous entrance point [5]. Reoperation via an inguinal incision may increase the risk of hemorrhage and cutaneous nerve or spermatic cord injuries because of difficult dissection through the fibrotic tissue [6].

Conversely, an anterior approach can be used to treat a recurrent inguinal hernia after a failed posterior approach according to the HerniaSurge Group and European Hernia Society (EHS) guidelines [7]. Sakamoto et al. reported the use of a “hybrid method,” i.e., explorative laparoscopy combined with open anterior repair, for a case of re-recurrent hernia after a failed anterior and posterior approach [8]. Although we performed a similar hybrid method on a patient who underwent primary repair via the laparoscopic transabdominal preperitoneal approach (TAPP), our technique is new to recurrent indirect hernia repair. We suggest that this is a useful method to reduce the incidence of surgical complications and re-recurrence. This study has been reported in line with the SCARE criteria [9].

## 2 Case presentation

**Fig. 1:**
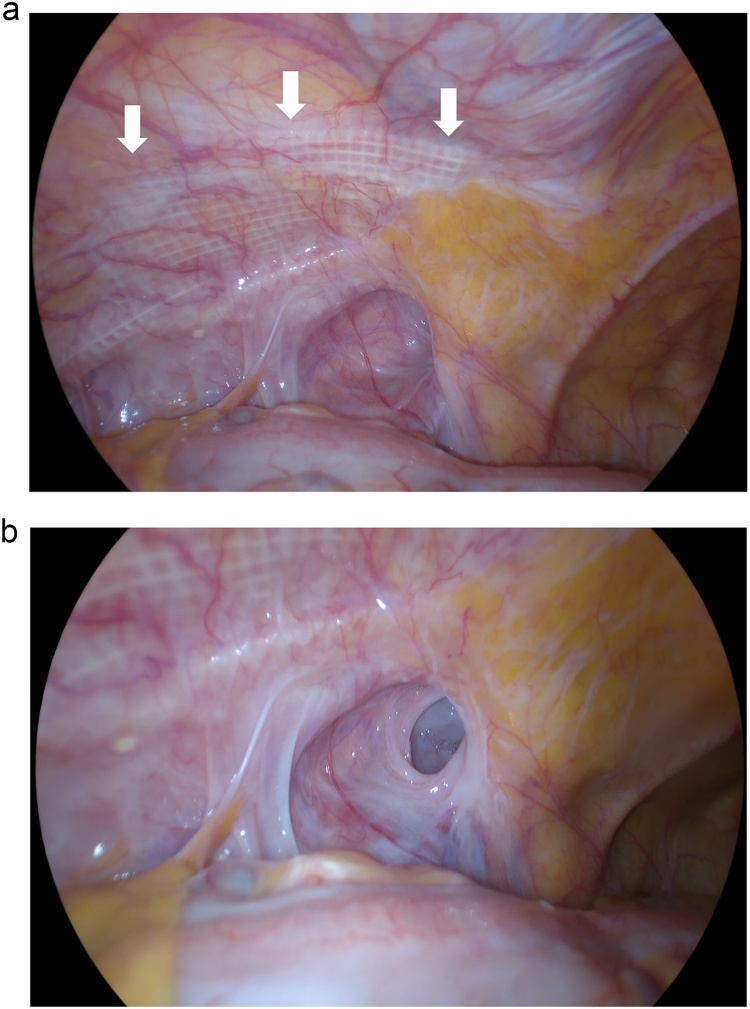
(a) Recurrent hernia orifice was confirmed inferior to the lower edge of the mesh (white arrow). (b) Close-up view of the hernia.

**Fig. 2:**
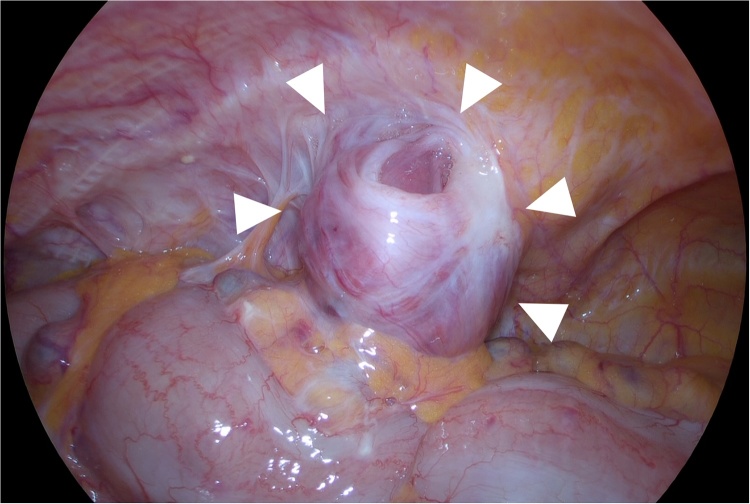
The plug (arrow head) appropriately covers the inguinal floor with the previous mesh through the observation of the abdominal cavity under laparoscopy.

A 74-year-old man underwent TAPP for a left indirect inguinal hernia (M2, according to EHS classification) with a Parietex*^TM^* (Medtronic plc. Dublin, Ireland) mesh measuring 13 × 9 cm. Recurrence was confirmed 5 years postoperatively. To diagnose the type of recurrence and clarify the location of the defect, the orifice was inspected using laparoscopy and a 2-cm indirect hernia was detected inferior to the lower edge of the mesh (Fig. 1a and b). Next, the skin was incised for the anterior open approach. The inguinal canal was opened in a standard manner, and the hernia sac was identified under increased pneumoperitoneum. After isolating the tissues surrounding the hernia sac, adequate space was secured in the preperitoneal cavity to insert a plug. Then, the plug was inserted into the defect following sac invagination under reduced pneumoperitoneum. An XL-sized PerFix*^TM^* (BD, Franklin Lakes, NJ, USA) plug (height 3.8 cm, diameter 5.1 cm) was fixed to the transverse fascia and the previous mesh with six interrupted stitches using absorbable sutures. Finally, the overlap was confirmed to sufficiently cover the myopectineal orifice, and the plug was inverted under the peritoneal membrane using laparoscopy with increased pneumoperitoneum (Fig. 2). The entire operation time was 1 h and 58 min. No complication was reported in the postoperative course nor was re-recurrence at 3 months postoperatively.

## 3 Discussion

Choosing the laparoscopic approach for the treatment of a recurrent inguinal hernia has the following advantages: apparent visibility of the hernia defect, the choice of an appropriate method, and reduction of complications and re-recurrence based on the previous procedure.

The re-recurrence rate was reported to be as high as 8.3% and 11.6% in the laparoscopic and anterior open repair groups, respectively [2], which was at least twice the occurrence rate compared to that of the primary inguinal hernia surgery [10]. Thus, all defects should be covered via minimum invasive routes without jeopardizing the structure. However, the nature of heterogeneous recurrent hernias and modification of previous surgical procedures with physical tissue reactions make planning the best approach before the operations difficult. Therefore, laparoscopy can be a useful method to provide significant information about the new anatomy and help select an optimal approach.

According to the HerniaSurge Group guidelines, a new, previously unused approach is more preferable than the previous route in the case of recurrent hernias. When recurrence occurs after abdominal or endoscopic preperitoneal operations, as in our case, an anterior approach is a safer and easier method. Moreover, this technique would be much more reliable if combined with explorative laparoscopy, which enables proper evaluation of the route to take and mesh for cover and confirms the perfection of overlap at the final step.

A PerFix*^TM^* plug was used in this case to cover the hernia defect on the dorsal side of the previous mesh. Some probable causes of recurrence are as follows: insufficient dissection and problems associated with the mesh such as a very small size, dislocation, or shrinkage. In our case, only the dorsal part of the previous mesh was found to be dislocated to the ventral side; however, it still overlapped most of the inguinal floor. Therefore, an additional large-sized plug without an on-lay patch would sufficiently fix the defect and reinforce the defect together with the previous mesh. As reported by Nienhuijs et al., the difference between mesh plug repair and Lichtenstein repair in terms of the recurrence rate was not significant [11]. Furthermore, a mesh plug that can prevent an ample dissection is beneficial because a recurrent hernia has a fourfold higher rate of leading to chronic neuralgia with moderate or severe chronic pain [12].

## 4 Conclusion

A hybrid method combining explorative laparoscopy and open anterior repair facilitates the choice of an optimal approach for recurrent hernia and may reduce surgical complications and the re-recurrence rate.

## Conflicts of interest

All authors declare that there is no conflict of interest.

## Sources of funding

This research did not receive any specific grant from any funding agency in the public, commercial, or not-for-profit sectors.

## Ethical approval

The institutional ethics committee determined that approval was not necessary for a case report.

## Consent

Written consent for the publication of this case report with accompanying images was obtained from the patient. The consent was written in Japanese for better understanding by the patient. The consent form will be provided to the editors of this journal on request.

## Author contribution

Study conception and design: Kikugawa, Tsujinaka.

Surgical team: Kikugawa, Tsujinaka, Tamaki, Takenami.

Image editing: Maemoto, Fukuda.

Critical revision of manuscript: Toyama, Rikiyama.

Final approval for submitting the manuscript: Kikugawa, Tsujinaka, Tamaki, Takenami, Maemoto, Fukuda, Toyama, Rikiyama.

## Registration of research studies

We have carefully read the Research Registry website. There is a statement ‘We do not register case reports that are not first-in-man or animal studies’ on the webpage.

Therefore, it is not necessary to register this case report.

## Guarantor

Shingo Tsujinaka, the corresponding author of this paper.

## Provenance and peer review

Not commissioned, externally peer-reviewed.
